# Impact of climate change on malaria transmission in Africa: A scoping review of literature

**DOI:** 10.4102/jphia.v16i1.1346

**Published:** 2025-08-28

**Authors:** Byron Chapoterera, Keshena Naidoo, Anesu Marume

**Affiliations:** 1 School of Nursing and Public Health, College of Health Sciences, University of KwaZulu-Natal, Durban, South Africa; 2 Department of Global Public Health, Faculty of Medicine and Health Sciences, University of Zimbabwe, Harare, Zimbabwe

**Keywords:** climate change, anopheles, plasmodium vector, global warming, rainfall, temperature

## Abstract

**Background:**

Climate change has significant health implications, disproportionately affecting Africa because of its limited adaptive capacity and socio-environmental vulnerabilities. Malaria, a climate-sensitive disease, is a major public health concern. Climate change influences malaria transmission by altering vector behaviour, parasite life cycles and environmental conditions.

**Aim:**

To identify, map and analyse evidence on the relationship between malaria and climate change in Africa.

**Setting:**

The study examined institutional and community-based studies conducted in the African region.

**Method:**

A systematic review of studies published between January 2010 and December 2024 was conducted across PubMed, Scopus, African Index Medicus and Embase using keywords related to climate change and malaria. Thematic analysis was applied to synthesise patterns and trends.

**Results:**

Ninety studies were included. Findings demonstrate that slight temperature increases significantly impact malaria transmission by accelerating mosquito vector and parasite development. Changes in rainfall patterns, whether excessive or diminished, affect mosquito breeding sites, leading to fluctuations in malaria incidence. Regional variations highlight the need for localised interventions.

**Conclusion:**

Climate factors are crucial in malaria prevalence and distribution in Africa. As climate shifts alter mosquito populations, traditional interventions, such as chemical spraying, may become less effective. Integrating ecological data into malaria control strategies ensures interventions remain effective under changing climatic conditions. Strengthening climate-responsive policies will be pivotal in malaria reduction and elimination efforts.

**Contribution:**

This review offers a comprehensive synthesis of climate–malaria interactions, providing insights for policymakers and researchers to develop climate-informed malaria control strategies tailored to Africa’s diverse ecosystems landscapes.

## Introduction

Malaria remains a significant public health challenge, with its burden disproportionately concentrated in sub-Saharan Africa (SSA).^1^ This region accounts for approximately 95% of all malaria cases and deaths worldwide, with the highest prevalence observed in Nigeria, the Democratic Republic of the Congo, Uganda and Mozambique.^1^ *Plasmodium falciparum*, the most common and lethal malaria parasite species in SSA, is primarily transmitted by *Anopheles gambiae* and *Anopheles funestus*.^2^ Other regions, such as South and Southeast Asia, Latin America and Oceania, also face substantial malaria burdens. However, the dominant parasite species and mosquito vectors vary, with *Plasmodium vivax* prevalent in Asia and *Anopheles darlingi* prominent in the Amazon basin.^2,3,4^

Climate change will likely worsen the global malaria burden, with SSA expected to face the most significant impact.^4,5^ Rising temperatures, shifting rainfall patterns and more frequent extreme weather events favour the spread of malaria-carrying mosquitoes, with a high impact in regions where populations lack access to effective prevention and treatment. Warmer climates can accelerate mosquito lifecycles, enhance the development of *Plasmodium* parasites, increase mosquito populations and extend transmission seasons.^6^ Altered rainfall patterns may create new breeding sites, while geographic shifts in suitable climates could introduce malaria to previously unaffected areas.^7,8^ Sub-Saharan Africa, already the epicentre of malaria, is particularly vulnerable because of its dependence on climate-sensitive agriculture and limited capacity to adapt to these environmental changes.^9,10^

Since the late 1980s, multiple malaria epidemics have been reported, significantly increasing morbidity and mortality in endemic regions and the East African Highlands. Regions such as the Eastern African Highlands, KwaZulu-Natal in South Africa, the Eastern Highlands of Zimbabwe, Gorongosa in Mozambique, Kenya, Uganda, Burundi, Tanzania, Eritrea, Ethiopia and Rwanda have all experienced surges in malaria cases.^11,12,13^ These epidemics have often been linked to changing climatic conditions, including abnormal heat, increased moisture and intensified El Niño events.^14,15,16^ El Niño refers to the warming of ocean surface temperatures in the central and eastern Pacific, which disrupts global weather patterns.^15^ Over the decades, these El Niño events have become more frequent and severe, amplifying conditions favourable to malaria transmission.^14,15^

From 1850 to 2005, global temperatures increased by approximately 0.7 °C and are projected to rise by 0.2 °C per decade.^17,18^ Such warming trends highlight the urgency of understanding the relationship between climate change and malaria to inform effective prevention and control strategies. Even in regions with strong malaria control measures, interannual weather changes have been shown to increase malaria incidence.^19,20^

The United Nations Development Programme (UNDP) defines climate as the average of weather patterns in a specific area over a prolonged period, typically 30 years or more.^21^ Climate change refers to long-term shifts in Earth’s climate, including warming of the atmosphere, oceans and land. As weather patterns evolve, malaria vectors may migrate to areas with more hospitable climates, exposing previously unaffected populations. Similarly, human settlements may shift to high-rainfall areas, increasing malaria risks. Conversely, in some cases, climate change could reduce malaria transmission by creating less favourable conditions for parasite and vector reproduction. Overall, temperature and rainfall are critical determinants of malaria onset, distribution, morbidity and mortality.

Indigenous knowledge systems have played a crucial role in helping local communities mitigate and adapt to climatic variability and its impacts on malaria transmission.^22^ These systems provide valuable insights that complement scientific models and enhance localised climate change mitigation and adaptation strategies. Furthermore, indigenous knowledge can detect climate-driven changes in malaria transmission dynamics, offering an additional layer of resilience in vulnerable regions.

This study seeks to identify, map and analyse evidence of the relationship between malaria and African climate change. Given the complexity of climate change health research, which spans multiple methodologies and focus areas, this study aims to provide actionable insights for health policymakers. The findings are intended to guide public health initiatives to reduce malaria morbidity and mortality in Africa while highlighting critical research gaps that need addressing.

## Methods

This review was guided by the methodological framework proposed by Arksey and O’Malley.^23^

### Search strategy

A systematic comprehensive search was conducted according to the scoping review protocol. The search was conducted across multiple databases, including PubMed, Scopus, African Index Medicus, Embase, Web of Science and Cumulative Index to Nursing and Allied Health Literature (CINAHL) to identify eligible studies. The search strategy was guided by the inclusion and exclusion criteria. A combination of keywords and Medical Subject Headings (MeSH) terms was employed to ensure the inclusion of relevant studies focusing on the relationship between climate change and malaria in Africa. The full search strategy for each database is provided in Online Appendix 2.

Additional studies were sourced through reference mining of the identified literature. The search encompassed literature published between January 2010 and December 2024. While the search was conducted in English, no language restrictions were applied to the identified literature.

### Eligibility criteria

Institutional and community-based studies were included if they reported malaria and climate change data in the African region and explicitly reported the observed or modelled effects of historical or future climate change on outcomes related to the distribution, dynamics or transmission of malaria, malaria cases in humans, the *Plasmodium* parasite or the mosquito vector. Papers were excluded if they were purely methodological or consisted of conference proceedings, preprints, abstracts without full text, literature reviews or case series.

### Data extraction

After searching the literature and removing duplicates, the studies were further filtered by reviewing their titles. Titles and abstracts of each study were independently screened by two postgraduate-level research assistants, with any disagreements resolved by the principal investigator. Subsequently, abstracts were reviewed in detail, and studies that did not meet the inclusion criteria were excluded. Full-text articles of the remaining eligible studies were then retrieved.

A data extraction tool (Online Appendix 1) was used to record key study details, including design, setting, sample sizes, climate variables, malaria trends, bibliographic information, methodologies, quantitative and qualitative findings and limitations. The selected studies were organised by author and publication date, full journal reference, research aims or questions, study population, age groups, geographic setting, study design, methodological approach to data analysis and reported outcomes. The included reports comprised quantitative, qualitative, mixed-methods and prospective studies.

### Quality assessment

The quality of the selected studies was systematically evaluated using the Mixed Methods Appraisal Tool (MMAT),^24^ a validated framework designed for the critical appraisal of studies employing qualitative, quantitative or mixed methods. The MMAT evaluates various dimensions of study quality, such as clarity of research questions, appropriateness of methodology, relevance of the data collection methods and robustness of the analysis. Each study was scored according to the MMAT criteria, allowing for a standardised assessment of methodological rigour. This process ensured the inclusion of studies that met minimum quality thresholds, thereby enhancing the reliability and validity of the synthesis. Studies with significant methodological limitations were flagged for cautious interpretation during analysis.

### Outcome measurement

This study aimed to evaluate the impacts of climate change on the malaria transmission cycle by examining key outcomes such as malaria incidence, prevalence and frequency of outbreaks. It also assessed changes in mosquito breeding patterns, including shifts in vector populations, geographic range and the length of transmission seasons. The study further explored how variations in temperature, rainfall and humidity, alongside adverse weather events such as El Niño, cyclones and prolonged droughts, influenced the lifecycle of malaria-carrying mosquitoes and the development of *Plasmodium* parasites. These factors collectively contribute to the changing dynamics of malaria transmission.

### Synthesis of findings

A thematic content analysis was employed to synthesise the findings from the included studies. This approach allowed for the identification of recurring patterns, significant concepts and emerging themes related to the effects of climate change on malaria transmission dynamics. The synthesis categorised findings into thematic areas, such as climate variables (e.g. temperature, rainfall), vector behaviours, parasite prevalence and human–environment interactions.

### Ethical considerations

Ethical clearance to conduct this study was obtained from the Medical Research Council of Zimbabwe (No. MRCZ/A/3168).

## Results

**FIGURE 1:**
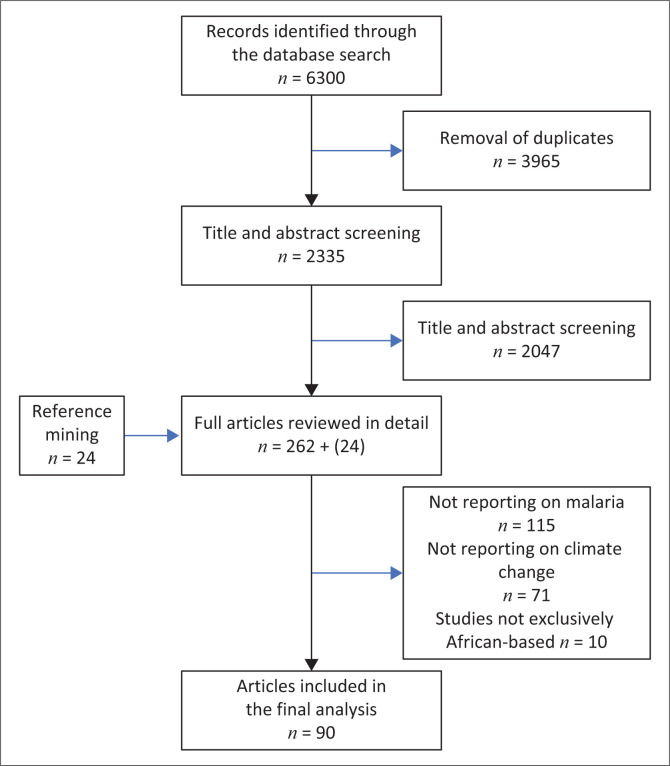
Preferred Reporting Items for Systematic Reviews and Meta-Analyses (PRISMA) Flow diagram for full-text selection for climate change and malaria.

The initial database search yielded 6300 articles. Following the removal of duplicates, 2335 studies remained for further screening. The titles of these studies were reviewed, resulting in a narrowed selection of 262 articles. To ensure comprehensive coverage, reference mining was conducted, identifying 24 relevant studies. This brought the total number of articles for full-text review to 286. Following this, 196 were excluded for either not reporting on either malaria or climate change or on the relationship between malaria and climate change and not being exclusively African based. A total of 90 studies were included in the final analysis (Figure 1).

### Quality assessment of identified studies

Of the 90 studies reviewed, 45% met all five quality criteria applicable to their respective designs, indicating a high level of methodological rigour (Online Appendix 1). Common strengths included clearly stated research questions, appropriate methodological approaches and the use of relevant data sources. However, some studies exhibited limitations, particularly in areas such as addressing potential sources of bias, clearly describing the sampling strategy and accounting for confounding factors. For some secondary data-based studies, they did not report the source of their dataset, while a few reported but did not outline data extraction processes clearly.

Overall, the quality of the included studies was deemed adequate to support a meaningful synthesis of the evidence on the links between climate change and malaria in Africa. The quality assessment results were used to inform the interpretation of findings, but no studies were excluded on the basis of quality alone.

### Characteristics of included studies

Of the 90 included studies, 31 were multinational. Of these, 15 assessed climate change and malaria across multiple regions of the African continent, three focused on various countries within SSA, six were specific to West Africa, six to East Africa and one to Southern Africa. Among individual countries, Ethiopia had the highest number of studies (*n* = 13), followed by Kenya (*n* = 8), Uganda (*n* = 6), South Africa (*n* = 4), Burundi (*n* = 4), Ghana (*n* = 4), Senegal (*n* = 3), Tanzania (*n* = 3), Malawi (*n* = 3), the Democratic Republic of Congo (*n* = 2) and one study each in the Gambia (*n* = 1), Niger (*n* = 1), Zambia (*n* = 1) and Eswatini (*n* = 1) Most of the studies (*n* = 37) used nationally representative datasets. This was followed by studies focusing on highland regions within selected countries (*n* = 7), while five studies (*n* = 5) used health facility-based datasets from specific districts or facilities. The remaining studies examined a single district, region or zone within individual or multiple countries (Table 1).

**TABLE 1:** Characteristics of identified studies.

Study	Country or region	Study setting	Timeframe	Study design	Human	Vector	Parasite	Climate
Abrha^25^	Ethiopia	Subnational	1950–2000 2040–2080	Laboratory-based study	NR	*An. arabiensis* and *An. funestus*	NR	Temp
Adu-Prah^26^	Ghana	National	1900–2009	Modelling	Malaria transmission	*F. An*. Spp. *NS*	*P. falciparum*	Temp, RF, RH, wind speed
Agyekum^27^	Ghana	Subnational	2016–2017	Laboratory research	NR	*An. gambiae* and *An. coluzzii*	NR	Temp
Akpan,^28^	Nigeria	National	1900–2010	Laboratory + Modelling	Malaria prevalence	*An. gambiae*	*P*. Spp. *NS*	Temp, RF
Alonso^29^	Kenya	Subnational	1970–2003	Modelling	Malaria epidemic	NR	NR	Temp
Artzy-Randrup^30^	Kenya, Uganda	National	10+ years	Modelling	Malaria immunity	*F. An*. Spp. *NS*	*P. falciparum*	Temp
Asefa^31^	Ethiopia	Subnational	NR	Cross-sectional study	Malaria cases	*F. An*. Spp. *NS*	*P. falciparum* and *P. vivax*	Temp, RH, sunshine, wind speed
Asori^32^	Ghana	Subnational	1970–2020	Modelling	Malaria prevalence	-	-	Temp, RF
Balikuddembe^33^	East Africa	National	1990–2019	Modelling	Malaria incidence	*F. An*. Spp. *NS*	*P*. Spp. *NS*	RF
Beck-Johnson^34^	CAR, Gabon, Mozambique, Zimbabwe	National	10+ years	Laboratory research	Malaria transmission	*F. An*. Spp. *NS*	*P*. Spp. *NS*	Temp, RF
Bomblies^35^	Niger	Subnational	NR	Modelling	Malaria transmission	*An. gambiae*	*P. falciparum*	Temp
Bouma^36^	Ethiopia	Subnational	1965–2015	Modelling	Malaria epidemic	NR	NR	El Nino
Boyce^37^	Uganda	Subnational	2012–2013	Modelling	Malaria transmission	NR	NR	Flooding, RF
Caminade^38^	Ghana, Mauritania, Niger, Senegal	National	1990–2010 2031–2050	Modelling	Malaria transmission	*An. gambiae* and *An. funestus*	*P. falciparum*	Temp, RF
Chaves^39^	Kenya	Subnational	1980–2009	Modelling	Malaria epidemic	NR	NR	Temp, RF
Chirombo^40^	Malawi	National	2004–2017	Modelling	Child malaria incidence	*F. An*. Spp. *NS*	*P. falciparum*	Temp, RF, RH
Chuang^41^	Eswatini	National	1985–2015	Modelling	Malaria incidence	*An. arabiensis*	*P. falciparum*	Temp, RF
Craig^42^	Africa (Selected)	National	NR	Modelling	NR	*An. gambiae* and *An. funestus*	*P. falciparum*	Temp, RF
Desoky^43^	Africa	National	NR	Secondary data analysis	NR	*An. gambiae*	*P. falciparum* and *P. vivax*	Climate, RF
Diouf^44^	Senegal	National	1910–2016	Modelling	Malaria transmission	*An. funestus*	*P*. Spp. *NS*	Temp, RF, RH
Diouf^45^	Senegal	National	2015–2100	Modelling	Malaria incidence	*F. An*. Spp. *NS*	NR	Temp, RF
Diouf^46^	West Africa (Selected)	National	1976–2005 2006–2065	Modelling	Malaria prevalence	*F. An*. Spp. *NS*	*P. falciparum*	Temp, RF
Drake^47^	Africa (Selected)	National	1950–2000 −>2050	Modelling	Malaria incidence	*An. arabiensis*	*P. falciparum*	Temp, RF
Egbendewe-Mondzozo^48^	Africa (Selected)	National	1990–2000 ™>2100	Modelling	Malaria incidence	*F. An*. Spp. *NS*	*P*. Spp. NS	Temp, RF
Emert^49^	Africa	Subnational	1960–2000 2001–2050	Modelling	Malaria cases	*F. An*. Spp. *NS*	*P. falciparum*	Temp, RF
Endo^50^	Ethiopia	Subnational	2006–2010	Modelling	NR	*F. An*. Spp. *NS*	*P. falciparum* and *P. vivax*	Temp
Ermert^49^	Africa (Selected)	National	1960–2000 2001–2050	Modelling	Malaria transmission	*F. An*. Spp. *NS*	*P. falciparum*	Temp, RF
Fall^51^	Senegal	National	2006–2099	Modelling	Malaria transmission	*An. gambiae* and *An. arabiensis*	*P. falciparum*	Temp, RF
Filho^52^	Africa (Selected)	National	2000–2018	Secondary data analysis	Malaria incidence	*F. An*. Spp. *NS*	P. Spp. NS	Temp
Glunt^53^	South Africa	Subnational	1990–2005	Modelling	NR	*An. arabiensis* and *An. funestus*	P. Spp. NS	Temp
Gone^54^	Ethiopia	Subnational	0ct 2011 – Feb 2012	Modelling	NR	*An. arabiensis, An. funestus, An. christyi, An. demeilloni, An. pharoensis* and *An. cinereus*	*P*. Spp. *NS*	Temp, RH
Hoshen^55^	Africa	National	1960–2000	Modelling	NR	*An. gambiae*	*P. falciparum*	Temp
Ishengoma^56^	Tanzania	Subnational	1981–2016	Modelling	individuals of 0–19 years	*Anopheles type NR*	*P. falciparum*	RF
Kimuyu^57^	Kenya	National	1950–2009	Modelling	Malaria prevalence	*F. An*. Spp. *NS*	*P*. Spp. *NS*	Temp, RF
Kleinschimdt^58^	Malawi	Subnational	NR	Modelling	Malaria in children	*F. An*. Spp. *NS*	*P*. Spp. *NS*	Temp, RF
Krsulovic^59^	Ethiopia	Subnational	1985–2007	Modelling	Malaria epidemic	NR	NR	Temp, RF
Kulkarni^60^	Tanzania	Subnational	2004–2014	Modelling	NR	*An. arabiensis*	*P. falciparum*	Temp, RF
Le^61^	Kenya	Subnational	2008–2013	Time series	NR	NR	NR	Temp, RF
Leedale^62^	East Africa	National	1980–2005	Cohort study	Malaria transmission	*F. An*. Spp. *NS*	*P*. Spp. *NS*	Temp, RF
Lindsay^63^	Gambia	National	10+ years	Modelling	Malaria transmission	*F. An*. Spp. *NS*	*P. falciparum*	Temp, RH
Lowe^64^	Malawi	National	2004–2011	Modelling	Malaria transmission	*An. gambiae* and *An coluzzii*	*P*. Spp. *NS*	Temp, RF
Lubinda^65^	Zambia	Subnational	2000–2016	Ecological case study	Malaria incidence	*F. An*. Spp. *NS*	*P*. Spp. *NS*	Temp, RF
Lyon^66^	Ethiopia	Subnational	1981–2014	Laboratory research	Malaria Epidemic	NR	NR	Temp, RF
Lyons^67^	Mozambique, SA, Zimbabwe	National	10+ years	Modelling	NR	*An. funestus* and *An. arabiensis*	*P*. Spp. *NS*	Temp, RH
Lyons^68^	South Africa	Subnational	NR	Modelling	Temperature effects	NR	NR	Temp
Martens^69^	Tropical Africa	National	NR	Modelling	Malaria Susceptibility	*An. macuipennis*	*P. falciparum* and *P. vivax*	Greenhouse effect IMAGE
Mattah^70^	Ghana	Subnational	1980–2012	Laboratory research	NR	*F. An. Larva*. Spp. *NS*	*P*. Spp. *NS*	Temp, RF, RH
Mbouna^71^	Cameroon	National	1985–2005	Modelling	Malaria prevalence	*F. An*. Spp. *NS*	*P. falciparum*	Temp
M’Bra^72^	Cote D’ivoire	Subnational	2004–2013	Modelling	Malaria cases	*F. An*. Spp. *NS*	*P*. Spp. *NS*	Temp, RF
Mordecai^73^	Africa	National	10+ years	Modelling	Malaria transmission	*F. An*. Spp. *NS*	*P. vivax*	Temp
Moukam Kakmeni^74^	West Africa (Selected)	National	2000–2050	Modelling	Malaria transmission	*An. funestus*	*P*. Spp. *NS*	Temp, RF, RH
Ndlovu^75^	South Africa	Subnational	NR	Modelling	NR	NR	NR	NR
Ngarakana-Gwasira^76^	Africa (Selected)	National (Not all countries)	1950–2000 2020–2039	Modelling	Malaria transmission	*F. An*. Spp. *NS*	*P. falciparum*	Temp, RF
Nigussie^77^	Ethiopia	Subnational	2012–2020	Modelling	Malaria transmission	*F. An*. Spp. *NS*	*P*. Spp. *NS*	Temp, RF
Nigussie^78^	Ethiopia	Subnational	NR	Quasi-experimental	Malaria transmission	NR	NR	Temp, RF, RH
Nkurunziza^79^	Burundi	National	1996–2007	Modelling	Malaria transmission	NR	*P. falciparum*	Temp, RF, RH
Nkurunziza^80^	Burundi	National	1996–2007	Modelling	Malaria incidence	*F. An*. Spp. *NS*	*P*. Spp. *NS*	Temp, RF, RH
Nkurunziza^81^	Burundi	National	1985–2005	Modelling	Malaria transmission	*F. An*. Spp. *NS*	*P*. Spp. *NS*	Temp, RF, RH
Nwaefuna^82^	Ghana	Subnational	NR	Modelling	NR	*An. coluzzii*	*P*. Spp. *NS*	RF
Nyawanda^83^	Kenya	Subnational	2008–2019	Modelling	Patients with febrille illness visiting Lwak Mission Hospital	*Anopheles type NR*	*P*. Spp. *NS*	Temp, RF
Ogega^84^	East Africa (Selected)	National	1977–2005 2008–2052	Modelling	Malaria transmission	*An. gambaie*	*P. falciparum*	Temp
Oliver^85^	South Africa	Subnational	NR	Laboratory research	NR	*An. arabiensis*	NR	Temp
Omumbo^86^	Kenya	Subnational	1979–2008	Modelling	Malaria debate	NR	NR	Temp
Ototo^87^	Kenya, Uganda, Tanzania	Subnational	1995–2010	Modelling	NR	NR	NR	Temp, RF
Paaijmans^88^	Kenya	Subnational	2005–2006	Modelling	Malaria epidemic	*An. gambiae*	NR	Temp, RH, wind speed
Paaijmans, 2010^89^	Africa	National	1960–2000	Experimental	NR	*An. stephensi*	*P. chabaudi*	Temp, RH
Paaijmans, 2014^90^	Kenya	Subnational	1981–2000 2046–2065	Modelling	Malaria transmission	*An. gambiae* and *An. arabiensis*	*P. falciparum*	Temp, RF
Panzi^91^	DRC	National	2001–2019 −>2036	Modelling	Malaria transmission	*F. An*. Spp. *NS*	*P. falciparum*	Temp RF, wind speed, RH
Panzi^92^	DRC	National	2001–2019	Modelling	Malaria transmission	*F. An*. Spp. *NS*	*P. falciparum*	Temp, RF, RH
Parham^93^	Tanzania	National	−>2080	Modelling	Malaria transmission	*An. gambiae*	*P. falciparum* and *P. vivax*	Temp, RF
Pascual^94^	East Africa	Subnational	1950–2002	Modelling	NR	*F. An*. Spp. *NS*	*P. falciparum* and *P. vivax*	Temp, RF
Protopopoff^95^	Burundi	Highlands	2002–2005	Modelling	NR	*F. An*. Spp. *NS*	*P. falciparum*	Temp
Riedel^96^	Zambia	National	2006	Modelling	Malaria in W&C	*F. An*. Spp. *NS*	*P*. Spp. *NS*	Temp, RF
Rodo^97^	Ethiopia	Subnational	1968–2007	Modelling	Malaria epidemic	NR	NR	Global warming
Ryan^98^	Africa	National	2000–2080	Modelling	Malaria transmission	*An. gambiae*	*P. falciparum*	Temp
Ryan^98^	Africa	National	−>2080	Modelling	Malaria transmission	*An. gambiae*	*P. falciparum*	Temp
Segun^99^	Nigeria	Subnational	2000–2013	Modelling	Malaria cases	*F. An*. Spp. *NS*	*P. falciparum* and *P. malariae*	Temp, RF, RH
Siya^100^	Uganda	Subnational	2011–2017	Modelling	NR	NR	NR	Temp, RF
Ssempiira^101^	Uganda	National	2013–2017	Modelling	Malaria incidence	*An. funestus*	*P*. Spp. *NS*	Temp, RF
Stern^102^	Burundi, Kenya, Rwanda, Uganda	National	1979–2009	Modelling	Malaria incidence	*F. An*. Spp. *NS*	*P. falciparum*	Temp
Taye^103^	Ethiopia	National	2004–2014	Modelling	Malaria prevalence	NR	NR	Temp, RF, RH
Tegegne^104^	Ethiopia	Subnational	-	Modelling	Malaria incidence	NR	NR	Temp, RF
Temel^105^	Ghana	National	2004	Case-control study	NR	*An. gambiae*	*p. falciparum*	Temp, RF
Tompkins^106^	Uganda	National	1926–1960	Modelling	Malaria transmission	*F. An*. Spp. *NS*	*P. falciparum*	Temp, RF
Tonnang^107^	Africa	National	1951–1990	Modelling	NR	*An. gambiae* and *An. arabiensis*	*P. falciparum*	Temp, RF, RH
Tourre^108^	Burkina Faso	Subnational	1983–2005 2010–2100	Modelling	Malaria epidemic	NR	NR	Temp
Woyessa^14^	Ethiopia	National	1991–2014	Modelling	Malaria incidence	*F. An*. Spp. *NS*	*P. falciparum*	Temp, RF
Yamana^109^	West Africa (Selected)	National	1980–1999 2080–2099	Modelling	Malaria incidence	*An. gambiae*	*P. falciparum*	Temp, RF
Yamana^110^	Niger	Subnational	2006	Modelling	NR	*An. gambiae*	NR	RH
Zhou^111^	Ethiopia, Kenya, Uganda	Subnational	1978–1998	Case-control study	Malaria cases	*F. An*. Spp. *NS*	*P. falciparum*	Temp, RF

F, female; *An., Anopheles*; NS, not specified; NR, not reported; Spp., species, Temp, temperature; RF, rainfall; RH, relative humidity; *P, Plasmodium*; SA, South Africa; DRC, Democratic Republic of the Congo.

Most studies (*n* = 81) used historical datasets or laboratory experimental data for malaria and climate outcomes, describing or analysing changes in either indicator. The remaining studies (*n* = 10) utilised historical data to estimate the potential effects of climate on malaria, either regionally or within individual countries. A significant proportion of studies (*n* = 66) employed modelling techniques to analyse the datasets, using various approaches such as Bayesian modelling, spatial modelling, time-series analysis and regression models. Other common modelling methods included machine learning techniques, agent-based modelling and dynamical systems modelling, which were used to simulate malaria transmission under different climate scenarios. Eight studies described various analytic study designs, including cohort studies (*n* = 2), cross-sectional studies (*n* = 2) and case-control studies (*n* = 4). These studies predominantly used secondary datasets or collected historical information during data collection. The remaining eight studies were experimental, with five conducted in laboratory settings and three using quasi-experimental designs. The laboratory studies were mainly related to manipulating the environment in which mosquitoes lived to see how climatic changes’ effect on climate change simulated real-world scenarios.

Nearly three-quarters (*n* = 67) of the studies examined the effect of climate on malaria in humans, with a similar proportion also considering the impact of climate on the female *Anopheles* mosquito (*n* = 74) or the *Plasmodium* parasite (*n* = 71). Among the studies focusing on malaria in humans, 26 analysed the effect of climate on malaria transmission, 11 investigated malaria incidence, 11 examined malaria immunity, eight explored malaria epidemics, six studied malaria cases, three focused on malaria in children and one addressed malaria in women and children. For studies examining the effect of climate on the mosquito vector, a majority (*n* = 41) did not specify the species. Among those identifying specific species, 17 considered *A. gambiae*, ten focused on *Anopheles arabiensis*, nine on *A. funestus* and two on *Anopheles stephensi*. One study investigated multiple species, including *Anopheles christyi, Anopheles demeilloni, Anopheles pharoensis, Anopheles cinereus* and *A. arabiensis*, while another study examined the climate’s effect on *Anopheles* larvae. Regarding the studies investigating the impact of climate on the malaria parasite, 27 did not specify the species, whereas 40 considered *P. falciparum*, seven focused on *P. vivax*, one on *Plasmodium chabaudi* and one on *Plasmodium malariae* (Table 1).

### Temperature changes, global warming and malaria transmission

Mosquito vector survival is limited at temperatures below 18 °C and above 32 °C, with most mosquitoes dying at temperatures exceeding 40 °C. The optimal temperature for *P. falciparum* incubation within mosquitoes is around 30 °C, while the most suitable temperature for mosquito vector development is approximately 22 °C.

A modelling study in Burundi found that a rise in minimum temperatures during the preceding month was strongly associated with increased malaria incidence, highlighting the strong relationship between temperature and malaria transmission.^79^ Similarly, a modelling study in Ghana demonstrated that temperature variations significantly impact malaria prevalence. Higher temperatures accelerate the development of both the parasite and mosquito vectors, increasing the risk of transmission.^32^ For instance, a 1 °C rise in temperature was shown to shorten the extrinsic incubation period of *P. falciparum* within mosquitoes, thereby enhancing transmission potential.^109^ Even modest temperature increases can substantially alter malaria dynamics in regions with endemic conditions. One of the included studies predicted that under global warming scenarios, malaria transmission might decline in regions already experiencing high temperatures.^47^

In contrast, other studies have shown that temperature increases could have significant consequences in cooler regions, particularly in the East African Highlands. A modelling study predicted that these highlands would face heightened malaria risks under 1.5 °C and 2 °C warming scenarios.^84^ The study also projected that previously malaria-free highland areas would likely experience increased transmission, making them vulnerable to outbreaks. Multiple studies in the Eastern Highlands reinforce this prediction, attributing recent regional malaria epidemics to rising temperatures.^61,68^ These studies argue that warming has enabled *Anopheles* mosquitoes to expand beyond their historical geographical range, colonising higher altitudes and increasing transmission potential in areas considered unsuitable for malaria.

### Extreme weather events and malaria transmission

Extreme weather events related to climate change include flooding and droughts that can affect the reproductive cycle of malaria vectors. Excessive rain can potentially wash away mosquito larvae and destroy breeding sites, leading to a temporary reduction in transmission. This was observed in several flood-prone areas of Eastern African countries such as Kenya, where surges in rainfall disrupted mosquito population growth.^33^ On the other hand, drought conditions, particularly in regions of East Africa, have been found to create unexpected breeding opportunities. During droughts, water scarcity often leads to the formation of isolated stagnant water bodies, such as puddles and shallow pools, which serve as ideal habitats for mosquito breeding. These habitats are less likely to be disturbed and can sustain mosquito populations, increasing the risk of malaria transmission.^33^ Moreover, the variability of rainfall patterns, exacerbated by climate change, adds another layer of complexity to malaria control efforts. Unpredictable shifts in rainfall intensity and distribution can lead to simultaneous flooding and drought risks within the same region, making it more challenging to anticipate and mitigate malaria outbreaks.

Four of the studies (*n* = 4) assessed the effect of extreme weather events on malaria transmission. Cyclones, tropical storms, flooding, heat waves and El Niño significantly influence malaria transmission dynamics, often creating conditions conducive to malaria transmission. This can be through disrupting ecosystems, destroying human settlements or increasing vulnerabilities.

Cyclones and tropical storms, characterised by heavy rainfall and flooding, create extensive breeding grounds for *Anopheles* mosquitoes. Floodwaters often lead to stagnant pools, ideal for mosquito larval development. At the same time, the displacement of communities during such events disrupts access to healthcare and preventive measures, increasing the risk of transmission. A study in Mozambique highlighted a surge in malaria cases following Cyclone Idai in 2019, with disrupted healthcare systems further compounding the burden. Similarly, tropical storms have been linked to malaria outbreaks in previously stable transmission areas in Madagascar.

On the other hand, heat waves can affect malaria transmission depending on local temperature thresholds. Extremely high temperatures may hinder *Plasmodium* development within the mosquito, reducing transmission in some regions. However, heat waves can accelerate transmission by shortening the extrinsic incubation period in areas where temperatures remain within the optimal range for both the parasite and mosquito vectors. In regions with limited cooling infrastructure, heat waves may also increase human exposure to mosquito bites during night-time outdoor activities.

El Niño events alter global weather patterns and have been associated with malaria epidemics, particularly in Africa. These events typically lead to increased rainfall in some regions and prolonged droughts in others, which can drive malaria transmission. For example, studies from East Africa have linked El Niño-induced rainfall with significant malaria outbreaks because of expanded mosquito breeding habitats. Conversely, drought conditions can create isolated water bodies, providing stable breeding sites that persist over time, as observed in semi-arid regions of Ethiopia.

Socio-economic vulnerabilities, such as inadequate infrastructure, displacement of communities and limited access to healthcare, compound the impact of these adverse weather events. Understanding the interaction between adverse weather events and malaria is essential for developing early warning systems and targeted interventions, particularly in regions prone to extreme weather.

Moreover, climate variability and extreme weather events such as El Niño and drought have been implicated in the recent re-emergence of malaria in regions like the East African Highlands. Studies suggest that the increased climate extremes since the late 1980s, including a higher frequency of El Niño events, have contributed to heightened malaria risk in these areas.^25^ Such extreme events not only influence malaria transmission by altering environmental conditions but also exacerbate socio-economic vulnerabilities, further complicating malaria control efforts.

### Human migration and environmental factors

The relationship between climate, socio-economic factors, the environment and malaria transmission is complex and context specific. A study in Gambia found that communities living in metal-roof houses were better protected from mosquitoes, with a higher proportion of mosquitoes being eradicated in households with metal roofs than those with thatched roofs.^63^

Climate change’s impact on agriculture, food security and animal rearing also creates a cascade of effects on malaria transmission. Many African communities rely on subsistence farming, and in efforts to mitigate climate impacts such as drought and flooding that threaten crops, land-use activities like deforestation and land degradation have been employed. Additionally, creating irrigation systems can increase mosquito breeding sites, heightening malaria risk. Deforestation in the African highlands and wetland conversion have allowed *A. gambiae* larvae to survive outside traditionally endemic areas. Deforestation has indirectly influenced climate change in Zambia, thus fostering conditions that promote malaria transmission.^112^

A study in Djibouti also found that rapid urbanisation, largely driven by climate change and globalisation, has facilitated the spread of *A. stephensi*, a mosquito species common and known to thrive mostly in urban environments.^113^ Similarly, in areas that were previously non-endemic, malaria outbreaks have often been associated with higher incidence and mortality rates, largely because of limited knowledge of malaria prevention.^97^

## Discussion

This review affirms the complex interplay between climate change and malaria transmission. Global warming and extreme weather events, such as flooding and droughts, can both positively and negatively affect malaria transmission.

The reviewed studies emphasise the need for localised and integrated malaria control strategies that address climatic and socio-economic dimensions. Enhanced surveillance systems, innovative vector control approaches and robust health infrastructure are essential to mitigate the impact of climate change on malaria. As climate change persistently modifies malaria risks, proactive and adaptive strategies are imperative to safeguard vulnerable populations and advance global malaria eradication objectives.

Most studies used statistical modelling to explore the relationship between climate change and malaria transmission. The use of diverse modelling approaches across studies provided valuable but varied insights. For instance, spatial analysis and seasonal autoregressive integrated models captured short-term variations in malaria incidence related to local climatic changes. In contrast, Bayesian models incorporating Markov Chain Monte Carlo simulations highlighted probabilistic outcomes and uncertainties. These models varied significantly in their methodological assumptions, data requirements and outputs, leading to different interpretations of the malaria–climate relationship.^114,115^ While in some cases they control for confounders, more often, models do not consider potentially influential factors such as urbanisation, land use changes and socio-economic determinants. These aspects could add depth to the understanding of malaria dynamics in the context of climate change; some are often unforeseen for studies that include future projections.

While some models underscored the immediate effects of rainfall and temperature on vector abundance, others projected long-term disease trends under various climate scenarios. Critically, these statistical approaches, while robust, present distinct limitations that influence their interpretative power. Spatial-temporal models, for example, excel in analysing localised impacts but may fail to capture broader, long-term trends because of their reliance on retrospective data. Conversely, predictive models like Box-Jenkins Seasonal Autoregressive Integrated Moving Average (SARIMA) or Autoregressive Integrated Moving Average (ARIMA) focus on future trends but often simplify the multi-dimensionality of climate and socio-economic factors, as they are primarily data driven and typically do not incorporate causal pathways without significant adaptation.^114,116^ The Liverpool Malaria Model provided unique insights by integrating meteorological and biological data; however, its complexity and reliance on extensive datasets make it less applicable in resource-constrained settings.^55^ These disparities underscore the need for harmonising modelling approaches to provide a holistic understanding of malaria dynamics under changing climatic conditions.

There were discordant findings reported in studies on the direction of malaria–climate change correlations. Some research identified a unidirectional correlation between climatological and epidemiological factors, some found a bidirectional relationship to a threshold point, and others found no relationship between malaria indicators and select climatological components. These discrepancies may result from methodological differences and the rapidly changing dynamics of malaria transmission. Analysis of the malaria survey’s geographical data revealed that spatial autocorrelation – the similarity in malaria prevalence numbers between nearby surveys – should be investigated.^40,79^

Changes in temperature, rainfall, humidity and human activities, contributing to climate change, such as deforestation and agricultural expansion, influenced malaria transmission. The relationship between climate change and malaria is complex and necessitates transdisciplinary research encompassing public health, anthropology, geography and other relevant fields. Additional variables, including sun exposure, wind speed and air pollution, could further elucidate the link between climate and malaria transmission.

The link between increased temperature and malaria transmission in Africa was inconsistently reported. Species-specific temperature responses may explain the variability in malaria transmission associated with rising temperatures.^40^ Contradictory findings regarding the temperature sensitivities of Anopheles mosquitoes highlight the need for consistent temperature monitoring to resolve these discrepancies. Regularly analysing daily temperature variations in predictive models is essential for improving malaria control and adapting to future climates.^43^

Climate data should be integrated into malaria control systems and decision-making processes involving stakeholders beyond health departments to enhance anti-malaria operations. Currently, meteorological services and health systems generate climate and health data independently resulting in fragmented approaches and missed opportunities for coordinated action.^91^ For example, in some regions of SSA, early warning systems for malaria outbreaks have underperformed because of poor data sharing between climate and health sectors. This disconnection requires technical solutions and institutional alignment, including cross-sectoral planning, data-sharing agreements and joint capacity-building.

Human activities, such as deforestation and swamp conversion in African highlands, also disrupt malaria prevention programmes. Governments must consider the environmental impact of land use changes and regulate agricultural and settlement activities accordingly. Malaria prevention programmes should adapt to ecological changes and the expansion of human settlements.

### Strength and limitations

While this review provides valuable insights into the relationship between climate change and malaria in Africa, several limitations should be acknowledged. Notably, the review did not explore certain potentially influential factors such as urbanisation, land use changes and socio-economic determinants in detail. These factors are intricately linked to climate variability and malaria transmission patterns, and their exclusion may limit the depth and contextual relevance of the findings. This omission partly reflects the scope and focus of the included studies, many of which did not explicitly examine these broader environmental and social determinants.

Variations in malaria testing algorithms such as rapid diagnostic tests versus microscopy complicate study comparisons. Climate data quality remains a concern, with significant data gaps reported. Other challenges include difficulty establishing causal mechanisms between climatological and epidemiological factors and needing to account for confounding variables like household spraying programmes, malaria prophylaxis and socio-demographic influences on malaria risk.

A notable gap in the reviewed studies was the lack of data on the impact of climate change on malaria-associated morbidity and mortality and the environmental effects of malaria prevention programmes. Future research should address these areas to inform environmentally responsible malaria strategies and enhance the effectiveness of interventions in the context of climate change. Increased preparedness requires resource allocation, ecosystem-based solutions and enhanced monitoring efforts. To develop comprehensive and adaptive solutions, stakeholders must prioritise integrating climate, ecological and social science research into malaria control initiatives.

The lack of reliable long-term environmental and temperature data in SSA hinders robust analysis of climate’s impact on malaria. Disparate methodologies and obsolete research techniques further exacerbate these challenges. Advanced data analysis, improved climate modelling and interdisciplinary approaches are urgently needed.

Finally, the review highlights a critical disconnect between malaria control efforts and climate change mitigation policies. The absence of integrated frameworks prevents holistic responses to the increasing malaria incidence in new regions. Transdisciplinary research and policy integration are essential to bridge these gaps and address the multifaceted challenges climate change poses to malaria dynamics.

### Recommendations

It is essential to adopt a multifaceted approach to address the challenges climate change poses to malaria transmission effectively. A key priority is the establishment of standardised frameworks for collecting and analysing climate and health data. Integrating these datasets can improve outbreak prediction and inform targeted interventions. Existing models, such as the WHO’s Climate Services for Health framework and the Integrated Disease Surveillance and Response (IDSR) strategy, offer practical entry points for aligning health systems with meteorological services through shared indicators and joint early warning systems. Enhanced surveillance systems that combine environmental and epidemiological data can significantly improve malaria control efforts by providing actionable insights. Tailored solutions that account for regional variations in climate and malaria dynamics are equally important. To ensure their effectiveness, interventions must be adapted to local conditions, such as temperature and rainfall patterns. For example, cooler highland regions experiencing warming may require proactive measures to prevent the spread of malaria into previously unaffected areas. At the same time, arid zones may benefit from strategies to manage breeding sites formed during droughts.

Innovative vector control methods also hold promise in mitigating the impact of climate change on malaria. Context-specific measures, such as eco-friendly housing designs that reduce mosquito entry and targeted management of mosquito breeding sites, can be highly effective. These approaches address current transmission patterns and build resilience against future climate variability. Furthermore, transdisciplinary research is critical to understanding the complex interactions between climate and malaria. Expanding the scope of studies to include underexplored factors such as wind speed, air pollution and socio-economic changes can refine predictive models and intervention strategies. Collaborative efforts across disciplines, such as public health, environmental science and urban planning, are necessary to develop comprehensive solutions. Finally, integrating malaria control efforts with broader climate adaptation and mitigation strategies is essential for long-term sustainability. Aligning policies across health, environmental and economic sectors will ensure that interventions are both practical and sustainable. By embedding malaria control within the broader context of climate change responses, governments and stakeholders can build more resilient systems to protect vulnerable populations and achieve lasting progress in malaria eradication.

Future research should prioritise study designs and analytical approaches that better elucidate causal mechanisms between climate variables and malaria transmission. Longitudinal studies, natural experiments and modelling approaches that account for temporal and spatial variability may be particularly useful in strengthening causal inference.

## Conclusion

The findings of the scoping review underscore the complex relationship between global warming, extreme weather events and malaria transmission. There was a notable lack of reliable long-term climate and malaria incidence data in most African countries affected by malaria. This hampers effective trend analysis and predictions regarding future risks.

However, the evidence of the significant impact of climate change on malaria transmission dynamics necessitates urgent climate adaptation strategies in the region to mitigate the impacts of changing malaria transmission dynamics. Suggested strategies include enhanced surveillance systems, particularly in previously low-risk areas like the highlands of East Africa and Ethiopia. Health systems should be more responsive to newly emerging malaria-affected regions by ensuring that diagnostic tools, antimalarial medications and preventive resources are available to these communities. Furthermore, while traditional vector control measures, such as mosquito nets and indoor residual spraying, remain central to malaria prevention, they should be augmented with environmentally focused solutions, such as sustainable water management practices that can address broader challenges posed by climate change. Chemical interventions alone were also shown to be insufficient in some regions, as changes in mosquito vector species because of climate change impaired the efficacy of insecticides. Of concern was the scarcity of data on indigenous knowledge systems to address the threats of climate change in African countries. Governments need to engage more with communities around the threat of climate change and malaria risks to develop robust and context-specific solutions to reduce deaths and hospitalisations from malaria.
